# Modified lateral gastrocnemius myocutaneous flap with extended anterior and/or inferior boundary

**DOI:** 10.1038/s41598-022-05093-2

**Published:** 2022-01-20

**Authors:** Ping Peng, Zhonggen Dong, Jianwei Wei, Lihong Liu, Zhaobiao Luo, Shu Cao, Qiang Xu, Lei Zheng

**Affiliations:** 1 Department of Orthopedics, The Second Xiangya Hospital, Central South University, No. 139 Renmin Road, Changsha, 410011 Hunan People’s Republic of China; 2 Department of Orthopedics, Hunan Provincial People`S Hospital (the First Affiliated Hospital of Hunan Normal University), Changsha, 410005 Hunan People’s Republic of China; 3 Department of Orthopedics. Zhuzhou Hospital Affiliated To Xiangya School of Medicine, Central South University, Zhuzhou, 412007 Hunan People’s Republic of China; 4 Department of Orthopedics, Henan Provincial People` Hospital, Zhengzhou, 450003 Henan People’s Republic of China

**Keywords:** Fracture repair, Orthopaedics

## Abstract

There is little information regarding the boundaries of the lateral gastrocnemius myocutaneous (LGM) flap. The aim of this study was to introduce the modified technique of the LGM flap with extended anterior and/or inferior boundaries and its anatomical basis. Five fresh lower limb specimens were perfused and radiographed. Between December 2003 and August 2018, 27 modified LGM flaps with extended anterior and/or inferior boundaries were raised in 27 patients to reconstruct the soft tissue defects over the middle and upper leg, knee, and lower thigh. Both the lateral popliteal cutaneous artery and musculocutaneous perforators from the lateral sural artery had rich linked arteries communicating with the chain-linked arterial network around both the posterolateral intermuscular septum and the sural nerve, and they also had rich transverse communicating arteries connecting with the perifascial arterial network overlying the anterior compartment in the upper and middle calf. Continuous fascial arterial networks were extended up to the level at the intermalleolar line. Twenty-three flaps survived uneventfully, 2 flaps displayed distal de-epithelialization, and 2 flaps (7.41%) developed partial necrosis. Osteomyelitis was cured successfully in all patients, and no relapse of infection was encountered during the follow-up period. Multiple feeder arteries are the arterial anatomic basis of the modified LGM flap. The modified LGM flap with extended anterior and/or inferior boundaries is feasible, and the modified flap with extended anterior boundaries is safe and reliable.

## Introduction

Soft tissue defects in the middle and upper leg, knee and lower thigh are often encountered by reconstructive surgeons. Although perforator flaps and vascularized free flaps have been reported to reconstruct these defects more frequently^1–3^, gastrocnemius muscular and myocutaneous flaps remain good alternatives for repairing these defects due to their relatively easy and quick procedure, large dimension, and reliable survival^4–6^. The medial gastrocnemius myocutaneous flap with a larger dimension and wider reach was applied more frequently to cover these defects^7,8^, while the lateral gastrocnemius myocutaneous (LGM) flap was used to resurface the defects when the defects were predominantly located in the lateral aspect of the regions mentioned above or when the medial gastrocnemius myocutaneous flap was unsuitable because its integrity was destroyed^9,10^. In 1978, according to a latex injection study that included fluorescence examination in vivo and ultimate flap survival in humans, McCraw et al.^11^ proposed that the boundaries of the LGM flap were as follows: the medial (posterior) margin was the midline posteriorly, the inferior margin was 10 cm above the lateral malleolus, and the anterior margin overlapped the fibula and could be expanded to carry skin over the lateral (but not the anterior) compartment.

Although some modified techniques of the gastrocnemius myocutaneous flap have been reported^12–14^, little information regarding the boundaries of the flap is available^15–17^. Cheng et al. reported successful medial gastrocnemius myocutaneous flaps with enlarged posterior and inferior boundaries in two patients^15^. In 2014, Agarwal et al. reported one LGM flap with an inferior boundary within 8 cm of the lateral malleolus after a delayed procedure^16^. In 2018, Panse et al. described that the inferior boundary of the LGM flap with the sural pedicle could be extended up to 5 cm proximal to the lateral malleolus^17^. There is no report discussing the extended anterior boundary of the LGM flap.

In clinical practice, we observed that some remnant skin (skin bridge) overlying the anterior compartment of the leg usually existed between the lateral border of the soft tissue defect and the anterior edge of the fibula (i.e., anterior boundary of the classical LGM flap). This skin bridge usually discommodes the design and transfer of the flap, and thus, it has to be removed or de-epithelized prior to flap transposition. If the skin bridge was joined to the classical LGM flap, the problem would be resolved. Meanwhile, the modified flap held a wider indication because of its expanded width and dimensions.

With a deeper understanding of the fasciocutaneous flap, perforator flap and vascular anastomosis (choke or true anastomosis)^1,2,18,19^, we presume that the modification of the LGM flap with expanded anterior and/or inferior boundaries is feasible and relatively safe. The aim of this study is to introduce the modified technique of the LGM flap with extended anterior and/or inferior boundaries and its anatomical basis, as well as report the outcomes of the modified flap for reconstructing defects.

## Results

### Anatomic observation

The lateral popliteal cutaneous artery arises from the popliteal artery, on average, 1.3 cm above the popliteal crease and then runs outward and down. The inferior lateral genicular artery exhibits three perforators 0–1 cm above the fibular head, one overlying the lateral compartment and two overlying the anterior compartment. One perforator from the circumflex fibular artery pierced the deep fascia over the lateral compartment 3.4 cm below the fibular head.

**Figure 1:**
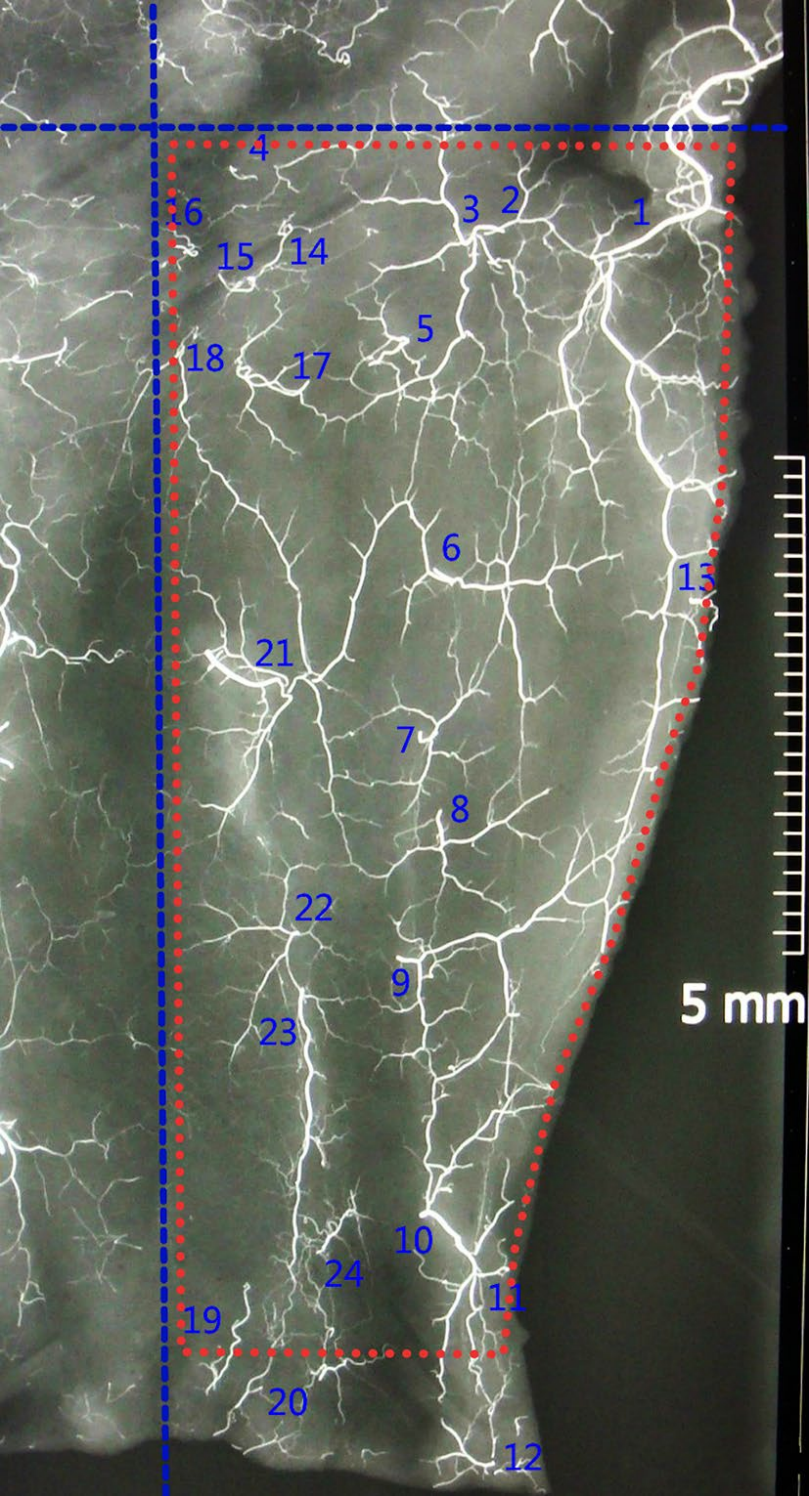
X-ray radiograph showing the arterial vasculature of the lateral leg integument. The integument was incised along the intermalleolar line and posterior midline. Transverse and longitudinal dotted lines (blue) represent the popliteal crease and anterior midline, respectively. The red dotted line displays the limit of the modified lateral gastrocnemius myocutaneous flap. The perforators arising from the inferior lateral genicular artery (2–4) were anastomosed with the perforators of the lateral popliteal cutaneous artery (1), circumflex fibular artery (5), anterior tibial recurrent artery (14, 15), anterior tibial artery (16–20), superficial peroneal artery (21, 22), and the arterial network around the superficial sural nerve (23). The perforators arising from the lateral popliteal cutaneous artery (1) were anastomosed with the perforators of the peroneal artery (6–11), lateral calcaneal artery (12) and lateral sural artery (13). The continuous arterial networks were extended up to the level of the intermalleolar line.

The arteriography showed the features as follows. The inferior lateral genicular artery anastomosed with the lateral popliteal cutaneous artery, perforators from both the upper anterior tibial artery and the circumflex fibular artery, and arterial network around the superficial sural nerve. Both the lateral popliteal cutaneous artery and musculocutaneous perforators from the lateral sural artery had rich linked arteries communicating with the chain-linked arterial network around both the posterolateral intermuscular septum and the sural nerve, and they also had rich transverse communicating arteries connecting with the perifascial arterial network overlying the anterior compartment in the upper and middle calf. (Fig. 1).

**Figure 2:**
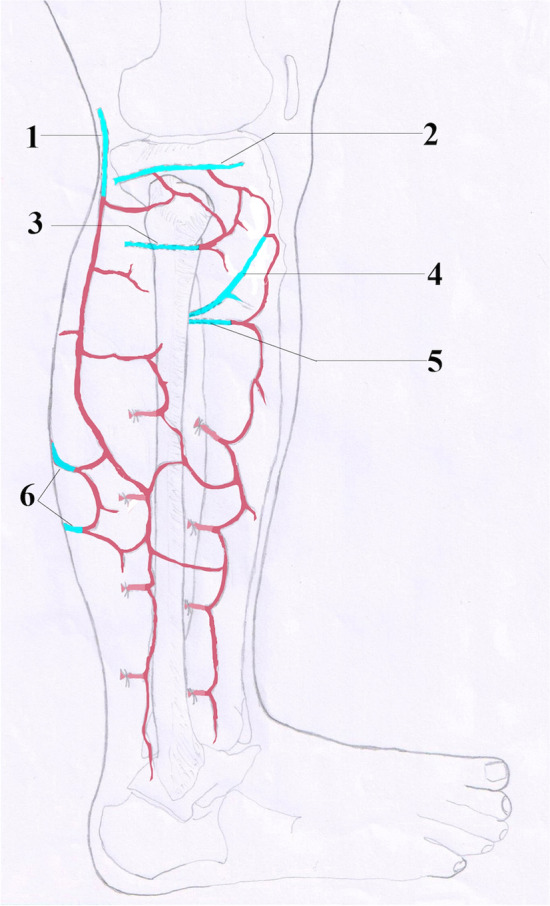
Schematic diagram of the arterial vasculature of the modified lateral gastrocnemius myocutaneous flap. The popliteal fossa lateral cutaneous artery (1) anastomoses with the perforators of the inferior lateral genicular artery (2), circumflex fibular artery (3), anterior tibial recurrent artery (4), anterior tibial artery (5) and lateral sural artery (6).

Continuous fascial arterial networks begin with the inferior lateral genicular artery over the anterior compartment, and both the lateral popliteal cutaneous artery and musculocutaneous perforators from the lateral sural artery over the posterolateral calf extend up to the level at the intermalleolar line. (Fig. 2).

### Clinical application of the modified flap

The patient and flap characteristics and the reconstruction outcomes are summarized in Table 1.

**Table 1:** Data on modified lateral gastrocnemius myocutaneous flaps with extended anterior and/or inferior boundaries.

Patients	Gender	Age (years)	Defect sites	Osteomyelitis classification#	Flap dimensions	Flap boundary (cm)			Outcomes
					(length × width, cm)	Anterior edge*	Inferior edge^	Superior edge&	
1	Male	35	Middle leg	II	24 × 11	6	> 10	13.2	Survival
2	Male	30	Upper leg	IV	25 × 13	3	> 10	1	Survival
3	Male	30	Middle and upper leg	II	20 × 10	5	> 10	4.5	Survival
4	Male	33	Upper leg	IV	23 × 12	6	> 10	1.7	Survival
5	Male	42	Middle and lower leg	IV	35 × 12	3	3	2.4	Partial necrosis
6	Male	42	Upper leg	III	22 × 14	7	> 10	4.4	Survival
7	Male	35	Middle leg	III	21 × 21	6	> 10	6.3	Survival
8	Female	36	Upper leg	III	23 × 13	4	7	2.4	Survival
9	Male	26	Middle leg	IV	20 × 12	4	7	4.4	Survival
10	Female	60	Middle leg	IV	24 × 8.5	2	5	6.2	Partial necrosis
11	male	42	Upper and middle leg	IV	22 × 7	0	5	10.3	Survival
12	male	35	Upper leg	IV	30 × 10	3	7	5.4	Survival
13	Female	42	Upper and middle leg	IV	26 × 11	4	> 10	3	Survival
14	Male	21	Middle and lower leg	IV	21.0 × 9.0	5	5	10.6	Survival
15	Male	38	Middle leg	IV	20 × 15	6	3	12.5	Survival
16	Male	30	Upper and middle leg	IV	30 × 12	4	5	4.5	De-epithelialization
17	Male	39	Knee and upper leg	III	26 × 8	2	10	2	Survival
18	Male	34	Knee and upper leg	III	20 × 11	2	12	1	Survival
19	Female	21	Knee and upper leg	III	23 × 9	1.5	10	3.5	Survival
20	Male	55	Middle and upper leg	IV	29 × 12	4.5	7	3	De-epithelialization
21	Male	40	upper leg	III	28 × 10	2	10	2	Survival
22	Male	56	Middle leg	IV	18.5 × 10	4	10	9.5	Survival
23	Male	57	Lower thigh and knee	III	26 × 8	2	> 10	4.9	Survival
24	Male	52	Upper and middle leg	IV	26 × 14	7	10	2.5	Survival
25	Male	45	Upper leg	III	20 × 10	7.5	> 10	2	Survival
26	Male	25	Middle leg	IV	24 × 12	5	10	7.2	Survival
27	Male	54	Middle leg	IV	18 × 14	6	> 10	10	Survival

^#^Cierny–Mader osteomyelitis classification system^22^.

*Distance exceeding the anterior edge of the fibula.

^Distance proximal to the tip of the lateral malleolus.

^&^Distance distal to the crease of popliteal fossa.

**Figure 3:**
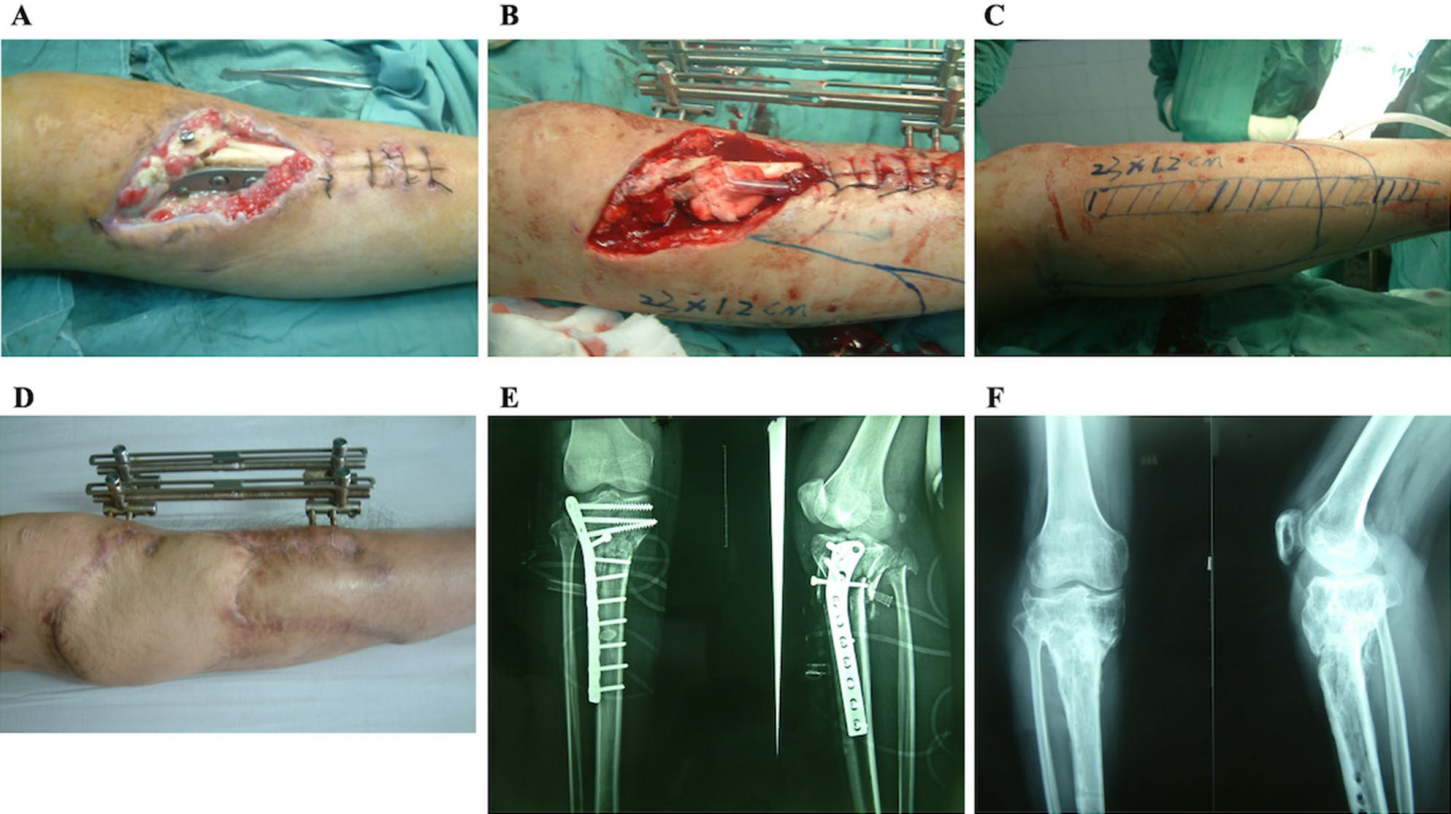
Soft-tissue defect over the upper leg combined with C-M type III tibia osteomyelitis in a 33-year-old male (**A**,**E**). A modified lateral gastrocnemius myocutaneous flap with anterior boundary extension was designed to reconstruct the defect after aggressive debridement (**B**,**C**). The dimensions of the flap were 23 cm × 12 cm, and the anterior extension exceeded the anterior edge of the fibula by 6 cm (**C**). The flap survived completely after 2 weeks, and the osteomyelitis was cured successfully. The fracture of the tibia healed successfully, and no infection relapse was observed during the 12-month follow-up (**D**,**F**). The patient regained ambulation without any assistance.

**Figure 4:**
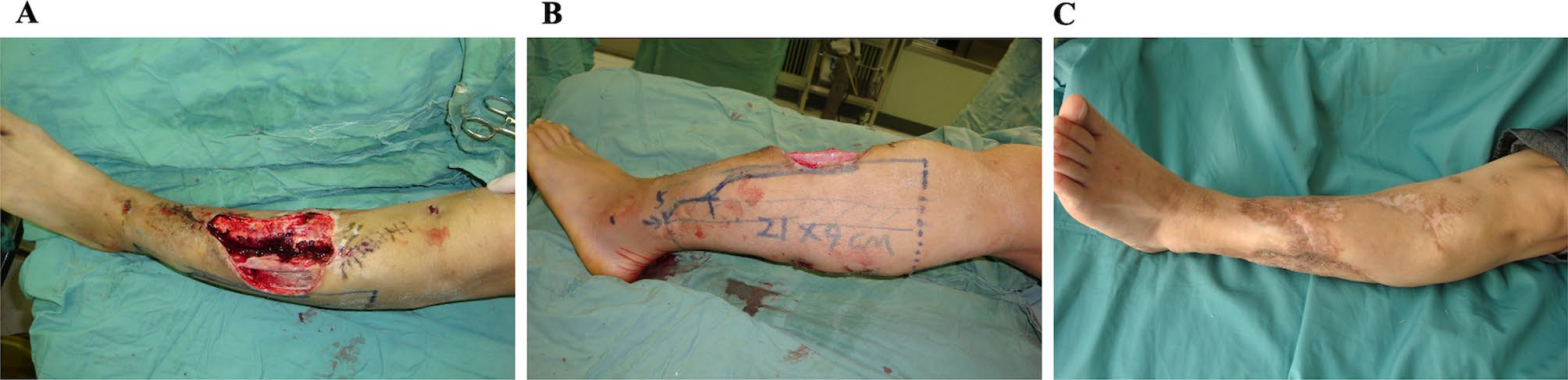
A 21-year-old male sustained C-M type IV tibia osteomyelitis with chronic ulcers on the middle leg over 10 years (**A**). A modified lateral gastrocnemius myocutaneous flap with anterior and inferior boundary extension was designed to reconstruct the defect after aggressive debridement (**B**). The flap survived completely after 2 weeks, and the infection was eliminated successfully. Excellent outcomes were achieved at 3 years postoperatively, and no recurrence of infection was observed during the follow-up period (**C**).

Among the 27 modified LGM flaps, 17 flaps had an anterior boundary extension (Fig. 3), 1 flap had an inferior boundary extension (5 cm above the lateral malleolus), and 9 flaps had both extensions (Fig. 4). The anterior edge of the 26 flaps with anterior extension exceeded the anterior edge of the fibula from 1.5 cm to 7.5 cm (mean 3.9 cm), and it reached the tibial crest in two flaps. Among the 10 flaps with inferior extension, the inferior edge of 2 flaps was located at 3 cm, 4 flaps at 5 cm, and 4 flaps at 7 cm above the tip of the lateral malleolus. The distance between the superior boundary of the flaps and the crease of the popliteal fossa ranged from 1.0 cm to 13.2 cm (mean 5.20 ± 3.60 cm). The length of the flap ranged from 18 to 35 cm (mean 23.80 ± 3.98 cm), the width of the flap ranged from 7 to 21 cm (mean 11.43 ± 2.76 cm), and the dimensions of the flap ranged from 18.5 cm × 10 cm to 35 cm × 12 cm.

There was no complete necrosis flap. Twenty-three flaps survived uneventfully, 2 flaps displayed distal de-epithelialization and 2 flaps (7.41%) developed partial necrosis (necrosis with length of 1.0 cm or more). All the partial necrosis flaps had both extended anterior and inferior boundaries, and the remnant wounds were successfully cured by employing other flaps (i.e., medial gastrocnemius myocutaneous flap or fasciocutaneous flap). The skin graft in the donor areas healed successfully in one stage, and no infection or other complications were encountered.

The follow-up procedures were performed in all 27 patients with a period ranging from 6 to 129 months (mean of 26.8 months). Osteomyelitis was cured successfully in 25 patients, and no relapse of infection, dehiscence or flap necrosis was observed during the follow-up period. Two patients with chronic osteomyelitis had infection recurrence at 1 and 12 months postoperatively, and the infection was eliminated through aggressive debridement and anti-infection treatment. No relapse occurred in the subsequent 10-year follow-up. All the patients regained stable ambulation without the assistance of the crutch or orthosis. Four patients had mild claudication without significant influence on their daily life. None of the patients complained about the aesthetic appearance.

## Discussion

Infection-free, durable, and adequate soft-tissue coverage were the preconditions to acquire stable function in patients with soft tissue defects and osteomyelitis in the middle and upper leg, knee, and lower thigh. Many techniques have been presented to achieve this goal, each of which has merits, demerits, and indications^1–10^.

The free flap could provide accurate and precise reconstruction for the defect, and excellent results were reported in the literature^1–3^. However, free flap surgery has several disadvantages, such as requiring expert surgeons in vascular separation and anastomosis, a longer operation duration, and sacrificing a main vascular trunk in most cases, which limits its wide application^23,24^. The keystone perforator island flap (KPIF) is one of versatile tool to reconstruct the defect over the lower extremity from the groin to the ankle, and it has many advantages, such as good aesthetic outcome, low complication rates, and efficient timeframe, et al.^25^ However, when the perforator vessels around the defect were damaged or become unreliable due to eroded by infection, other flaps may be required.

The myocutaneous and muscular flaps have robust vascular distribution and large dimensions, which contribute to the control of infection and can resurface tremendous defects simultaneously. Since the gastrocnemius myocutaneous flap was described in the 1970s by Mccraw^26^, it has been widely employed to repair soft tissue defects in the scenario mentioned above^5–7^, and many improved techniques and flap styles have been reported^12–17^. Many publications^5–14^ have reported its applications for reconstructing the defects mentioned above, and excellent outcomes have been achieved. However, little information can be acquired regarding the reliable limit of the LGM flap.

The lateral sural artery originates from the popliteal artery and enters the lateral gastrocnemius muscle at the proximal site of the muscle. It divided dendritic multistage vascular branches in the muscles and raised many musculocutaneous perforators to the subcutaneous tissue, which nourished the lateral gastrocnemius muscle and skin overlying the muscle^11,26^. The classical LGM flap was considered to be supplied by the lateral sural artery. Mccraw et al.^11^ proposed the boundary of the traditional LGM flap based on the injection of barium sulfate-red latex mixture in a single main artery, intraoperative fluorescein angiography and clinical observation. The boundary might also include the lateral popliteal cutaneous artery, but the authors did not mention this. Our anatomical study results showed that the blood supply of the modified LGM flap was multisource. Not only the lateral sural artery but also the lateral popliteal fossa cutaneous artery, the perforators of the lateral inferior genicular artery and anterior tibial recurrent artery contributed to the survival of the modified flap. When the superior boundary of the flap was lower, the perforators from the circumflex fibular neck artery, anterior tibial artery, superficial peroneal artery, posterior tibial artery and/or peroneal artery might also be included in the flap.

Our anatomical study results showed that the lateral popliteal cutaneous artery and musculocutaneous perforators from the lateral sural artery had rich transverse communicating arteries connecting with the suprafascial arterial network overlying the anterior compartment in the upper and middle calf. These observations provided an anatomical basis for the feasibility of extending the anterior boundary of the LGM flap to the surface of the anterior compartment of the lower leg and furthest to the tibia crest.

In the clinical scenario, it is usually observed that some remnant skin exists between the lateral border of the defect and the anterior edge of the classical LGM flap. To facilitate flap transfer, the remnant skin had to be removed or de-epithelized, which increased the complexity of the operation and was a waste of the tissue. The modification of the LGM flap with extended anterior and/or inferior boundaries not only made the design and operation of the flap easier and quicker but also increased the width, effective length, and dimension of the flap, thus expanding its indications. This technique allowed rapid, durable, and reliable coverage of the defects mentioned above without sacrificing a major nerve or vessel of the extremity. The results of this study showed that all 17 LGM flaps with extended anterior boundaries and inferior boundaries more than 10 cm above the tip of the lateral malleolus survived completely. This result suggested that the extension of the anterior boundary of the LGM flap was feasible and reliable. To the best of our knowledge, this is the first study to discuss the extended anterior boundary of the LGM flap.

Agarwal et al. reported one surviving LGM flap with an inferior boundary within 8 cm of the lateral malleolus^16^. Panse et al. described that the inferior boundary of the LGM flap with the sural pedicle could be extended up to 5 cm proximal to the lateral malleolus^17^. However, the number of reported cases was few, and no anatomical evidence was displayed to prove the reliability of the flaps with extended inferior boundaries.

Our anatomical study discovered continuous fascial arterial networks beginning with the inferior lateral genicular artery over the anterior compartment, and both the lateral popliteal cutaneous artery and musculocutaneous perforators from the lateral sural artery over the posterolateral calf could extend to the level of the intermalleolar line. This result provided an anatomical basis for the feasibility of extending the inferior boundary of the LGM flap. In this case series, there were ten flaps with inferior boundary extension, and four of them with inferior borders 7 cm above the lateral malleolus survived completely. The inferior boundary of the four flaps was located 5 cm above the lateral malleolus, and one flap developed partial necrosis. Partial injury to the integrity of the flap and severe infection might be the causes. Two flaps with the inferior border extending to 3 cm above the lateral malleolus and partial necrosis occurred in one flap that was too large (35 cm × 12 cm). These results suggested that the modification of the extended inferior boundary of the LGM flap was relatively feasible and reliable with proper control of the indications.

The important characteristics of osteomyelitis are recurrent infection and resistance to treatment. The recurrence rate of osteomyelitis is an important index to evaluate the effectiveness of surgery, and it is 4.3% ~ 28% as reported in the literature^27,28^. In this series, all defects were associated with various degrees of osteomyelitis. Recurrence of infection was observed in two cases (7.41%), which was controlled with redebridement and application of antibiotics, and no relapse was encountered during the 10-year follow-up period. All the fractures healed successfully, and most patients resumed preoperative activities. However, a flap that had a slightly bloated appearance and "cat ear" deformity, and a donor area that needed a skin graft were weaknesses.

The anatomical observation and clinical application in this study were limited due to few specimens. Further studies with larger sample sizes are required to verify the reliability and effectiveness of the modified lateral gastrocnemius myocutaneous flap with extended anterior and/or inferior boundaries.

## Conclusion

Multiple feeder arteries, including the lateral sural artery, lateral popliteal cutaneous artery, and inferior lateral genicular artery, are the arterial anatomic basis of the modified lateral gastrocnemius myocutaneous flap with extended anterior and/or inferior boundary. The modified lateral gastrocnemius myocutaneous flap with extended anterior and/or inferior boundary is feasible, and the modified flap with extended anterior boundary is safe and reliable.

## Methods

### Anatomical observation

Five fresh lower limb specimens were obtained from five patients (aged 25–47 years) with recurrent malignant bone tumours in the upper two-thirds of the femur (including three malignant fibrous histiocytomas and two osteosarcomas) in our department who underwent amputation at the level of the upper femur (n = 2) or hip joint (n = 3). This study was conducted in accordance with the Declaration of Helsinki and received approval from the Ethics Committee of the Second Xiangya Hospital Central South University. All patients involved in this study gave informed written consent to participate.

The specimens were irrigated with heparinized saline through the femoral artery immediately after amputation and injected with a barium sulfate-red latex mixture (10 ml red latex, 20 ml normal saline and 27 g barium sulfate)^20^. The specimens were refrigerated at 4 °C for 24 h. Then, four specimens were incised at the anterior midlines and one at the posterior midlines of the lower extremities. The integuments of the limbs were dissected at the subfascial plane. All the perforators were encountered and their source vessels were marked during the dissection. The dissection was performed until the entire integument was stripped. Radiographs of the harvested integuments were taken by a digital X-ray machine (ball tube height, 120 cm; exposure time, 0.80 s; and current, 125 mA; SHIMADZU, Japan)^21^.

### Patients and methods

Between December 2003 and August 2018, 45 lateral gastrocnemius myocutaneous flaps were raised in 45 patients to reconstruct the soft tissue defects over the middle and upper leg, knee, and lower thigh. In this study, 27 LGM flaps with extended anterior boundaries, extended inferior boundaries or both were included, and LGM flaps with traditional boundaries were excluded. Among 27 patients, there were 23 males and 4 females, with an average age of 38.5 (range, 21 to 57) years old. This retrospective study was conducted in accordance with the Declaration of Helsinki and received approval from the Ethics Committee of the Second Xiangya Hospital Central South University. Written informed consent was acquired from all patients.

The duration between injury or diagnosed osteomyelitis and the operation ranged from 1 month to 10 years (mean, 17.1 months), and the aetiologies of the defects were trauma-related events in 24 patients and chronic osteomyelitis with sinus or chronic ulcer in 3 patients. All the defects were combined with various degrees of osteomyelitis, and no defects were caused by diabetes mellitus or peripheral arterial disease. The size of the defect ranged from 5 cm × 4 cm to 22 cm × 12 cm.

### Surgical procedure

#### Management of recipient site

The patient was placed in the lateral position with the injured limb upwards and not fixed firmly so that the position of the patient could be readily changed when necessary during the operation. A sterile rubber tourniquet was placed over the thigh without exsanguination, which could facilitate the operation under bloodless conditions and skin graft harvest from the thigh.

Aggressive surgical debridement was performed to eliminate all the infected and nonviable soft tissues and bones. If a fracture existed, the internal fixation was replaced with the external fixator. When Cierny–Mader (C–M) type I–II osteomyelitis^22^ existed, the infected surface bone was removed with an osteotome, and drilling of the bone to release the pressure was performed when necessary. When osteomyelitis is C–M type III–IV, cortical fenestration should be performed to eliminate all the infected tissues and inflammatory granulation in the medullary canal. The infected tissues were collected for bacterial culture and drug sensitivity tests. Normal saline (at least 6 L) and hydrogen peroxide (500 ml) were alternately used to irrigate, and vacuum-assisted closure was adopted to cover the wound.

#### Design of the modified LGM flap

The limits of the modified LGM flap were as follows: the medial (posterior) boundary was the midline posteriorly, the anterior boundary could arrive at the tibial crest, the inferior boundary was 3 cm above the tip of the lateral malleolus, and the superior boundary was the crease of the popliteal fossa (Fig. 1).

The modified flap was designed within the boundary mentioned above. The posterior boundary of the flap was delineated along the midline posteriorly, the anterior boundary ran along the lateral border of the defect and its curved extension line, the superior boundary was at the level of approximately 2 cm above the upper edge of the defect, and the inferior boundary was outlined at the level of approximately 5 cm below the lower edge of the defect (Figs. 3B, 4B,C). A paper template was employed to simulate the flap for covering the defect without tension and could be readily adjusted if necessary before flap harvest.

#### Flap elevation

The posterior boundary of the flap was incised first to identify the small saphenous vein and sural nerve, which were the landmarks of the posterior midline of the calf and should be protected in situ. The aponeurosis between the lateral and medial gastrocnemius at the inferior segment was identified, incised, and separated. The loose space between the lateral gastrocnemius and soleus aponeuroses was identified and separated bluntly with the index finger. The lateral gastrocnemius was cut off at the musculotendinous junction. After that, the inferior and anterior boundaries of the flap were incised, and the flap was elevated in the subfascial or submuscular plane from bottom to top. Dissection stopped at the superior boundary of the flap. The link between the skin flap and muscle should be protected meticulously to protect the perforator from the muscle to the skin. The flap was then transposed to cover the defect, and the donor site was resurfaced with a skin graft.

### Consent for publication

Written informed consent was obtained from all participants.
